# Human Y chromosome copy number variation in the next generation sequencing era and beyond

**DOI:** 10.1007/s00439-017-1788-5

**Published:** 2017-04-04

**Authors:** Andrea Massaia, Yali Xue

**Affiliations:** 1 National Heart and Lung Institute, Imperial College London, London, SW7 2AZ UK; 2 Wellcome Trust Sanger Institute, Wellcome Genome Campus, Hinxton, CB10 1SA UK

## Abstract

The human Y chromosome provides a fertile ground for structural rearrangements owing to its haploidy and high content of repeated sequences. The methodologies used for copy number variation (CNV) studies have developed over the years. Low-throughput techniques based on direct observation of rearrangements were developed early on, and are still used, often to complement array-based or sequencing approaches which have limited power in regions with high repeat content and specifically in the presence of long, identical repeats, such as those found in human sex chromosomes. Some specific rearrangements have been investigated for decades; because of their effects on fertility, or their outstanding evolutionary features, the interest in these has not diminished. However, following the flourishing of large-scale genomics, several studies have investigated CNVs across the whole chromosome. These studies sometimes employ data generated within large genomic projects such as the DDD study or the 1000 Genomes Project, and often survey large samples of healthy individuals without any prior selection. Novel technologies based on sequencing long molecules and combinations of technologies, promise to stimulate the study of Y-CNVs in the immediate future.

## Introduction

**Fig. 1:**
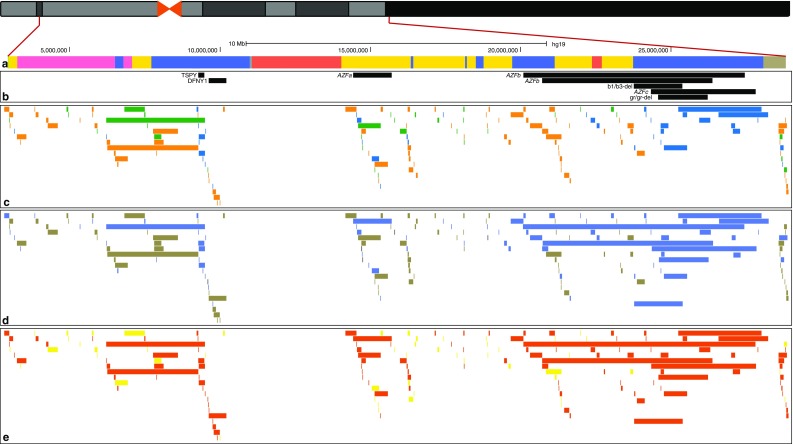
Summary of CNVs on the human Y chromosome. **a** Male-specific euchromatic region of the Y chromosome. The Y-specific unique region is shown in *yellow*, the X–Y transposed region in *red*, Y-specific repeats in *blue*, heterochromatic segments in *purple* and other regions in *grey*. **b** Medically important Y-CNVs. **c** CNVs discovered from population studies. Deletions are shown in *orange*, duplications in *green*, and deletions/duplications in *blue*. **d** Y-CNV mutation events inferred from the available data. Single events are shown in *yellow*, recurrent events in *blue* and unknown ones in *dark grey*. **e** Y-CNVs associated with segmental duplications or other repeats are shown in *dark orange*, and non-repeat-associated ones in *yellow*

The Y chromosome, here referring to its haploid, male-specific portion (MSY), is a unique segment of the human genome. It is non-essential for the life of an individual but required for male sexual differentiation, and evidence for its role in human biology beyond male reproduction is growing (Bellott et al. 2014). Its functional uniqueness is matched by its structural complexity: the human Y is rich in repeated elements and segmental duplications, which cover ~35% of its length (Skaletsky et al. 2003). While polymorphisms for presence and absence of repeated elements are common in the rest of the genome (Conrad et al. 2010; Mills et al. 2011; Sudmant et al. 2015), only two such polymorphisms, the insertion of an *Alu* element, that in the phylogeny identifies haplogroup DE (Hammer 1994), and the insertion of a LINE-1 element in a subgroup of haplogroup O (Santos et al. 2000) are known in the Y chromosome. Nevertheless, repeated elements are tied to other classes of genomic rearrangements: they are believed to be directly involved in one of the proposed mechanisms for structural rearrangements (non-allelic homologous recombination, NAHR) and their frequent presence near putative CNV breakpoints has been described in the Y chromosome (Poznik et al. 2016) (Fig. 1), as in the rest of the genome (Conrad et al. 2010). Intuitively, the abundance of repeats is a possible cause (Redon et al. 2006), but also a plausible consequence of frequent structural rearrangements. For instance, the palindromes in ampliconic regions (Skaletsky et al. 2003) show a high arm-to-arm sequence similarity, which is proposed to be maintained by frequent gene conversion events (Rozen et al. 2003): this may have the effect of preserving important, fertility-related genes from decay over evolutionary timescales by both reducing the accumulation of deleterious mutations when coupled with purifying selection, and also by facilitating the fixation of potential beneficial mutations when coupled with positive selection (Betran et al. 2012).

**Fig. 2:**
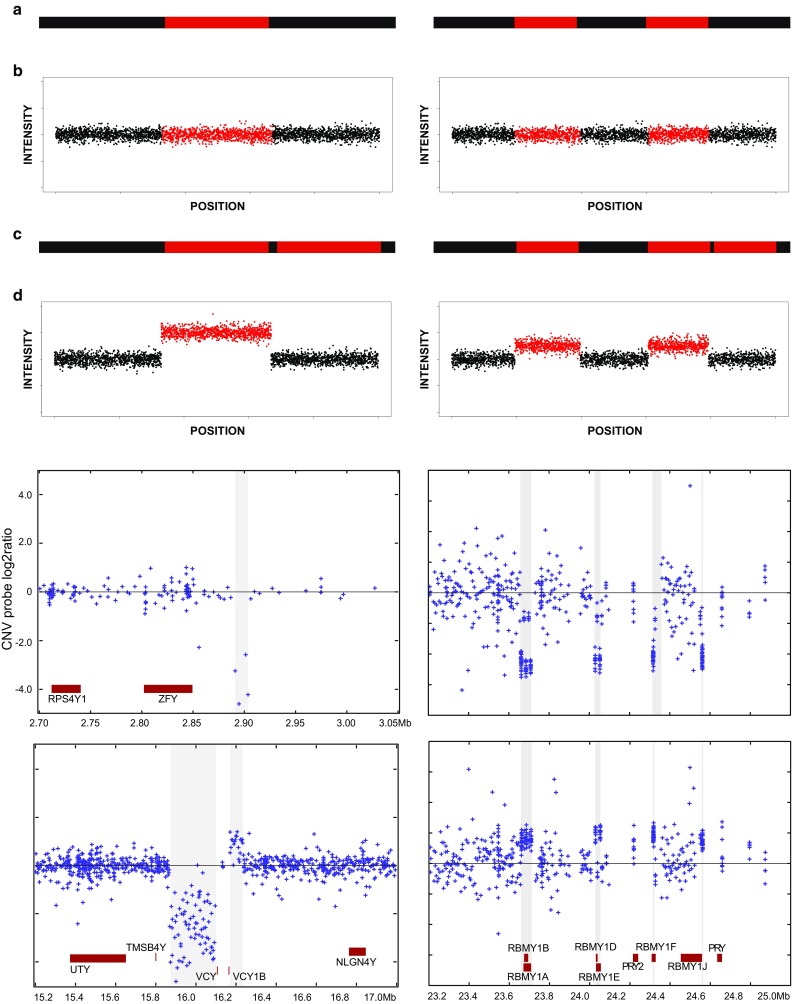
Shadowing effect for intensity data. The *top half* shows schematic representations of CNVs and the corresponding intensity data plots. **a** A unique region (*left*) or duplicated region (*right*) in the reference genome is shown in *red*. **b** Corresponding *plots* showing the intensity signal for each probe, here represented by a single *dot*, on the Y axis, and the position for the probe on the X axis. **c** A hypothetical duplication of the unique region (*left*), and of one of the copies of the duplicated region (*right*). **d** The unique region will show a stronger increase in signal (*left*), as compared to the duplicated region (*right*); in the duplicated region, moreover, the increase will be detected in both reference copies, as the method is unable to distinguish between them. The *bottom half* shows real examples for both CNVs in unique regions (*left*) and in a repeated region showing the shadowing effect (*right*) (from Wei et al. 2015). On the *right*, the *RBMY* gene copies all show co-ordinated intensity changes

While repetitive sequences may facilitate structural rearrangements, they also make their detection harder: emblematically, when the sequence of the Y chromosome was published in 2003, *Nature*’s cover described it as “a genetic hall of mirrors”. Most current detection methods are tailored to the diploid genome, and their prior expectations about copy number may not be adequate to the haploidy of the Y chromosome. Long, highly similar inter- and intra-chromosomal multicopy sequences make reference-based methods unreliable, making it difficult to map sequencing or intensity data correctly, and to univocally assign observed variation to a specific region, an effect defined as “shadowing” (Wei et al. 2015) (“Box 1” and Fig. 2). Despite these difficulties, several regions of the chromosome are well known to be prone to specific rearrangements (Jobling 2008), and these have continued to be investigated by focused studies in the past few years. The abundance of information about these regions mostly depends on some specific features, which historically led to the discovery of these variants, such as the effects on male fertility of azoospermia factor (*AZF)* loci (Vogt et al. 1996), the high and hypervariable copy number of the *TSPY* gene (Tyler-Smith et al. 1988), or the failure in sex testing caused by *AMELY* deletions (Santos et al. 1998). Wider studies of Y-CNVs have been scarce until recent years, and genome-wide CNVs investigations touched the Y chromosome only marginally. The pioneering study by Redon et al. (2006), which employed a combination of BAC arrays and comparative genomic hybridization to build the first CNV map of the human genome, reported over 250 variants on the Y chromosome. What looked like a promising start for Y-CNVs turned into a notable exception as subsequent genome-wide CNVs studies largely ignored the Y chromosome, either because they were carried out in females (Conrad et al. 2010) or simply because they reported a very small number of Y-CNVs, if any (Mills et al. 2011; The 1000 Genomes Project Consortium 2012). In the 1000 Genomes Project Phase 3, only six structural variants on the Y chromosome were described by genome-wide analyses (Sudmant et al. 2015; The 1000 Genomes Project Consortium 2015). In the latest structural variation (SV) data release by the Genomes of the Netherlands Project (Francioli et al. 2015; The Genome of the Netherlands Consortium 2014), which also produced a dedicated study of structural variants (Hehir-Kwa et al. 2016), only 4556 out of 1,851,571 structural variants (less than 0.25%) were mapped to the Y chromosome. Such studies focused on the diploid genome; it is only recently that similar high-throughput, unbiased studies have focused on the Y chromosome.

In this review, we first discuss the methodologies used in the past and present and some potential developments in the future for studying Y-CNVs, then the observations we have gained so far from the targeted studies, and finally the chromosome-wide studies.

## Methodologies for CNV studies

Several methods have been employed in Y-CNVs studies, and the choice is mostly driven by the resolution and power offered by each method, and the intrinsic features of the information produced. A summary of the methods presented below is given in Table 1.

**Table 1:** Summary of CNV detection and follow-up methods used currently or in the past in Y chromosome studies

Method	Resolution*	Throughput	Analysis procedure	Application
Karyotyping	5 Mb	Low	Visual inspection	Genome-wide detection
Interphase FISH	100 kb–1 Mb	Low	Visual inspection	Validation, detection of complex structures
Fibre-FISH	10 kb–1 Mb	Low	Visual inspection	Validation, detection of complex structures
BAC array CGH	100 kb	High	Intensity detection and processing	Genome-wide detection
Oligo array CGH	500 bp	High	Intensity detection and processing	Genome-wide detection, validation
Short read WG sequencing	1 bp	High	Read mapping and variant calling, de novo assembly	Genome-wide detection
Long read WG sequencing	1 bp	High	Read mapping and variant calling, de novo assembly	Genome-wide detection
qPCR, paralogue ratio test (PRT)	200 bp	Medium	Fluorescence detection	Validation, copy number quantification
Digital droplet PCR (ddPCR)	200 bp	High	Fluorescence detection	Validation, copy number quantification
Sequence-tagged site (STS)	200 bp	Medium	Electrophoresis: band presence/absence	Validation, targeted assay
Breakpoint PCR	1 bp	Low	Electrophoresis: band presence/absence	Validation and refinement

* Resolution indicates the (approximate) minimum size of variants each method is able to detect, except when a range is given, where the maximum is also indicated. Note that not all methods are suitable for all CNVs; further details are given in the text

Cytogenetics allows direct observation of the alternative structures generated by structural rearrangements. However, cytogenetic methods have low throughput due to the laboriousness of the technology that often requires cell culture and high levels of skill in implementation and interpretation, making it inconvenient to analyse more than a handful of samples at once; the resolution falls in a wide range depending on the specific method. Karyotyping allows the detection of aneuploidies and rearrangements down to ~5 Mb in size; it has been used to observe Y aneuploidies ranging from zero copies, as in Turner syndrome (Legro 2012), up to four copies (Paoloni-Giacobino and Lespinasse 2007), and provides a validation method to confirm mosaicism (Poznik et al. 2016). It is notable that the first evidence for an *AZF* locus on the Y (Tiepolo and Zuffardi 1976) and age-related somatic loss of Y (Jacobs et al. 1963) were from cytogenetic analyses. Higher resolution can be achieved with fluorescence in situ hybridization (FISH) techniques, which can be performed on interphase nuclei or metaphase chromosomes, in a setup similar to non-fluorescent karyotyping, but also on linearized chromatin fibres. Fibre-FISH allows for greater detail, ranging from ~100 kb when using BAC-clone derived probes, down to a few kilobases when using custom probes; moreover, using multiple probes can reveal inversions, a class of rearrangements otherwise difficult to detect. FISH-based methods can be used as validation in large studies (Poznik et al. 2016), but can also be used as the main investigation method when targeting specific variants, such as the change in size of the long arm heterochromatin block (Repping et al. 2003, 2006).

PCR-based methods are also, generally speaking, low-throughput, as the PCR technology produces short-range information (with a single reaction usually limited to less than 10 kb) and is then impractical to employ alone in genome-wide or chromosome-wide studies. At the same time, however, PCR is easily scalable to a large number of reactions: a recently described application, droplet digital PCR (Hindson et al. 2011) can process up to 2 million reactions in a single workflow. PCR approaches are then ideal in the screening of large cohorts for a limited number of specific variants. For instance, the study by Rozen et al. (2012) used PCR-based STS detection to assess the *AZFc* structure in over ~20,000 individuals. PCR also allows multiplexing, enabling the analysis of more than one region at the same time: this has been exploited to design the *AMELX/Y* sex test (Sullivan et al. 1993), but also to design clinical tests for *AZF* microdeletions (Vogt and Bender 2013). Simultaneous reactions can also be employed to directly count the number of members in a gene family by real-time quantitative PCR (Kumari et al. 2012). Real-time PCR has also been used to design a test for the Y chromosome in free foetal DNA in maternal blood (Boon et al. 2007), thanks to its high sensitivity and specificity. Above all, Sanger sequencing of PCR products makes it possible to reach base-pair resolution. This allows, for instance, targeting and validation of breakpoints and inference of mutational mechanism from the surrounding sequence. Together with the high scalability, this makes PCR a gold standard validation method, even in large-scale studies (Mills et al. 2011).

Microarray technologies infer CNVs by interpreting intensity signals, rather than detecting them directly. Compared to cytogenetics and PCR-based methods, microarray-based methods can produce a notably higher amount of information. For instance, the Illumina Infinium Core-24 Kit analyses up to ~600,000 markers, promising a throughput of 2800 samples per week. Different technologies exist, with resolution from ~100 kb in BAC-clone based arrays (Redon et al. 2006), to ~500 bp with high-resolution oligonucleotide probes (Conrad et al. 2010), including SNP arrays. Array-based methods have been used in many large-scale CNV studies, including both genome-wide and Y-specific studies, either as the main data source (Conrad et al. 2010; Johansson et al. 2015; Redon et al. 2006; Wei et al. 2015), or to validate discoveries from sequencing (Mills et al. 2011; Poznik et al. 2016). Besides the advantages of high data output and easy scalability, however, microarrays present a critical limit, in that they are based on sequence similarity between probe and target. This feature is especially problematic in the presence of long, nearly identical repeats, such as those on sex chromosomes. In these instances, array-based methods (and specifically, technologies based on shorter probes such as SNP arrays and array CGH) will not be able to assign an unequivocal signal to each of the repeated units; a change in copy number at one repeat will be reported as a much smaller change, of the same sign (increase or decrease), at each one of the repeats, making it impossible to tell which one is actually mutating. (“Box 1” and Fig. 2).

Next generation sequencing (NGS) is now established as the prime data-generation method in genomics, and Y chromosome CNV analysis is no exception. NGS offers high throughput comparable to, and even higher than, microarray-based methods; it can potentially achieve base-pair resolution, and indeed has been employed recently as the main data source to study CNVs in the Y chromosome (Espinosa et al. 2015; Poznik et al. 2016) as well as in the rest of the genome (Hehir-Kwa et al. 2016; Mills et al. 2011; Sudmant et al. 2015). It should be noted, however, that the Y chromosome genomic context amplifies NGS’s intrinsic limitations. First, sequencing data analyses require mapping to a reference sequence, a step which is confounded by the highly repetitive nature of the Y (Jobling 2008). Uncertainty in mapping produces the “shadowing” effect mentioned earlier and shifts the focus of data analyses, explaining the abundance of computational methods developed to handle NGS data in CNV studies (Pirooznia et al. 2015); methods which are, however, usually tailored to the diploid genome, and may require additional care when applied to the MSY. Second, while sequencing can theoretically reach base-pair resolution, technical limitations such as low depth (median read depth of 4.3×) can preclude the identification of smaller variants; for instance, the smallest CNV identified by Poznik et al. (2016) on the Y chromosome was 2.5 kb. Furthermore, most NGS platforms rely on short reads, thus producing short-range information that can fail to detect complex rearrangements, including copy neutral events such as inversions and translocations, and produces limited information about breakpoints. In this respect, the development of long read sequencing technologies such as PacBio (Rhoads and Au 2015) or Oxford Nanopore (Laver et al. 2015) appears promising. Long-range information is also produced by 10X Genomics, through short read sequencing of individually barcoded long molecules, or “linked-read sequencing” (Zheng et al. 2016). The preprint by Spies et al. (2016) showed how this method can be used to resolve complex structural variants. The same group published a similar study using a similar technology of “synthetic long reads” developed by Illumina, named TruSeq (Bishara et al. 2015). These long read sequencing methods will also provide “gold standard” CNV calls for calibrating other calls.

## Targeted Y-CNV studies

The *AZF* loci on the long arm are among the most active rearrangement hotspots in the human genome, and are some of the most studied because of their medical relevance (Repping et al. 2006). The three loci (*AZFa*, *AZFb* and *AZFc*, with *AZFb* and *AZFc* partially overlapping) were identified when their deletion was associated with azoospermia or severe oligozoospermia (Vogt et al. 1996). In recent years, *AZF* rearrangements have been surveyed in samples from many populations, including the Chinese (Lu et al. 2009, 2011; Yang et al. 2015; Zhang et al. 2013; Zhu et al. 2016), Dutch (Noordam et al. 2011), Europeans (Krausz et al. 2009), Jordanians (Khabour et al. 2014), Indians (Ambulkar et al. 2015; Kumari et al. 2015) and Iranians (Alimardanian et al. 2016; Motovali-Bashi et al. 2015), benefiting from the development of novel typing methods (Motovali-Bashi et al. 2015; Saito et al. 2015; Zhou et al. 2015; Zhu et al. 2016). The effects of *AZF* rearrangements on fertility have been summarised in a study by Lo Giacco et al. (2014), which presented data collected from diagnostic infertility testing over several years. Among 806 sterile males from several populations (~73% Spanish), the authors report 27 males with complete *AZF* deletion, including six showing abnormal karyotype and 21 with Y chromosome microdeletion. The authors also conducted a case–control study of partial *AZFc* deletions, showing that *AZFc* gr/gr and b2/b3 deletions (Repping et al. 2003), discussed further below, where significantly more frequent among sterile males than controls.

Among the *AZF* loci, *AZFc* region stands out for its complexity (Kuroda-Kawaguchi et al. 2001) and for the variety of alternative structures (Lu et al. 2011; Repping et al. 2006; Yang et al. 2015). A large study was conducted on over 20,000 males from India, Poland, Tunisia, the United States and Vietnam, assaying sequence-tagged sites (STSs) that mark different microdeletions (Rozen et al. 2012). This survey found that 3.7% of the sample had one of four deletions (gr/gr, b1/b3, b2/b3 and b2/b4) among those previously described in the region (Kuroda-Kawaguchi et al. 2001; Reijo et al. 1995; Repping et al. 2002, 2003). Individual frequencies for the assayed deletions varied widely across populations, from 15% for the gr/gr deletion in Vietnamese males, to 0.043% for the b1/b3 and b2/b4 deletions in Polish individuals, and down to the undetectability of the P5/P1 and P4/P1 (*AZFbc*) deletions. Moreover, the frequency of gr/gr and b2/b3 varied significantly across populations, with the latter probably due to differences in the prevalence of haplogroup N1 samples, in which the deletion is fixed (Fernandes et al. 2004). Rozen and colleagues also observed that haplogroup R1a appeared enriched in gr/gr deletions in the Polish population and in b1/b3 deletions among the samples from the United States. Finally, they estimated population frequency and contribution to severe spermatogenic failure (SSF) for the gr/gr, b1/b3, and b2/b4 deletions, concluding that about 8% of cases of SSF could be explained by either the 3.5 Mb b2/b4 deletion, which is rare (0.043% frequency) but has a strong effect (145-fold risk increase), or the more common 1.6 Mb gr/gr deletion (2.2% frequency), which doubles the risk of SSF (Rozen et al. 2012). From this and other studies we see how the gr/gr deletion appears to have a major impact on fertility, due to its combination of frequency and risk increase. This result also emerges from several meta-analyses (Navarro-Costa et al. 2010; Stouffs et al. 2011; Tuttelmann et al. 2007; Visser et al. 2009). The different penetrance and variable effect on the risk of spermatogenic failure observed for *AZFc* deletions might also depend on co-occurring compensatory duplications, hinting that besides causing an imbalance in gene dosage, *AZFc* rearrangements might affect fertility by altering the non-coding structure of the region (Yang et al. 2015).

Another highly active rearrangement hotspot lies on the *p* (short) arm of the chromosome, where the *TSPY* gene is present in a large and highly variable number of copies, organized as an array of 20.4 kb long elements (*TSPY* major), plus a single copy of the gene (*TSPY* minor) located more distally (Skaletsky et al. 2003). The copy number of TSPY has been observed to vary widely across population samples, up to 64 copies (Mathias et al. 1994; Oakey and Tyler-Smith 1990; Repping et al. 2006; Tyler-Smith et al. 1988); intraspecific variation comparable to that in humans has also been recently observed in gorillas (Tomaszkiewicz et al. 2016). *TSPY* organization represents 70% of the differences in functional gene number between the Y chromosome of humans and chimpanzees (Hughes et al. 2010): while the overall *TSPY* copy number, including inactive copies, is similar, chimpanzee Y chromosomes carry three arrays rather than two, and most of the copies are pseudogenes. A more detailed human–chimpanzee comparison (Xue and Tyler-Smith 2011) suggested that an ancestral array in the human–chimpanzee common ancestor might have undergone expansion in the human lineage and multiple duplications in the chimpanzee lineage; moreover, human–chimpanzee sequence comparison pointed to positive selection as a likely mechanisms of evolution for *TSPY* (Xue and Tyler-Smith 2011), implying a selective advantage in having multiple copies of the gene. In humans, *TSPY* is expressed exclusively in testis (Schnieders et al. 1996), and its copy number has been shown to have an effect on spermatogenesis, although results on this are discordant (Giachini et al. 2009; Nickkholgh et al. 2010; Vodicka et al. 2007): perhaps too few and too many copies both increase the chance of spermatogenic failure. All of these medically important Y-CNVs are shown in Fig. 1.

*TSPY* arrays are also involved in a different form of structural variation. Non-allelic homologous recombination (NAHR) between *TSPY* major and *TSPY* minor can cause a deletion over 3 Mb long (Jobling et al. 2007; Santos et al. 1998), which removes several genes. Among these, *AMELY* is probably the most studied due to its importance in forensics. Multiplex PCR coamplification of portions of different length of the *AMELX* and *AMELY* gene pair (106 and 112 bp, respectively) is routinely used for sex identification (Sullivan et al. 1993); however, *AMELY* deletions (including but not limited to *TSPY*-mediated deletions) or point mutations at primer binding sites cause the test to identify such males as females (Tozzo et al. 2013). The wide usage of *AMELX/Y* testing has then led to the discovery of different rearrangements involving *AMELY*, with frequencies ranging from around 0.02% (Chen et al. 2014; Mitchell et al. 2006; Xie et al. 2014) to 8% (Santos et al. 1998), and ranging in length between 304 bp (Mitchell et al. 2006) and 4 Mb (Jobling et al. 2007). Systematic studies of these variants are scarce; such rearrangements appear rare, except in South Asia where they reach ~2% (Thangaraj et al. 2002). Despite the co-deletion of *PRKY*, *TBL1Y* and *PCDH11Y* in the larger events (Jobling et al. 2007), and reciprocal duplications (Murphy et al. 2007; Wei et al. 2015), no phenotypic effect has been described so far besides the aforementioned sex testing failure. Novel methods for sex testing have been proposed, which often integrate or replace the *AMELX/Y* test with *UTX/Y*, *SRY* or microsatellite typing (Cadamuro et al. 2015; Santos et al. 1998; Tozzo et al. 2013).

## Chromosome-wide studies

The recent escalation in large-scale genomics has produced a wealth of information that can be employed in CNV studies. A good example of this, and also of how several studies on copy number variation have excluded the Y chromosome from their analyses, is the study published by Johansson et al. (2015). This study used SNP-array data to analyse a total of 1718 males from 13 previously published projects which excluded the Y chromosome from their analyses, although it included 510 males from phase 3 of the HapMap project (International HapMap C 2003). The full dataset covered several different populations, and given the multiple origins of the data, included samples gathered for the purpose of analysing conditions as diverse as schizophrenia, bipolar disorder, developmental disorders, high-altitude adaptation, cancer prostate, motor neuron disease, and colorectal cancer. Some highly variable regions on the chromosome were covered incompletely (*AZFc*) or not covered at all (*TSPY*) by the SNP-array probes. Nevertheless, Johansson and colleagues were able to identify 25 Y-chromosomal CNV patterns in their sample set, with an excess of duplications over deletions. Some of the variants identified were novel, and three variants were extremely rare, being identified in one individual each. The authors reported a significant association of ten variants with one or more haplogroups, which might represent a signature of rare events, likely to happen once in the Y phylogeny. The authors also tested the association of CNVs with the conditions present in their dataset, but did not detect any significant association.

Large projects such as the DDD project (The Deciphering Developmental Disorders Study 2015) and the 1000 Genomes Project (The 1000 Genomes Project Consortium 2010, 2012, 2015) are powerful enough to alone enable researchers to investigate copy number variation across the whole chromosome, as demonstrated by several studies in recent years. In one study published in 2015, CNVs across the whole MSY were investigated in 411 apparently healthy males from the UK, using an array CGH design that had been employed in the DDD study; SNP-array data were used to validate the CNVs discovered in a subset of individuals (Wei et al. 2015). After merging overlapping CNVs called in individual samples into CNV events (CNVEs) and manual curation, 22 curated CNVEs (curCNVEs) were identified. Raw, individual events ranged in length from less than 1 kb to over 3 Mb, the latter corresponding to the *AMELY* duplication described above. More than half the events were observed in just one individual, but six had frequency higher than 5%, up to 26% (107/411 individuals). Deletions (relative to the reference used) were more abundant than duplications, but this was heavily influenced by two curCNVEs that were deleted in 76 and 68 individuals, respectively. None of the ten curated CNV events present in more than one individual was specific to a single Y haplogroup, implying recurrent mutational events for all of them. The curated set of variants covered 24 protein-coding genes, some of which had already been extensively investigated for CNVs, like the *AZFc* region, *TSPY* and *AMELY* CNVs discussed above. In addition, a previously undocumented partial duplication in the *AZFa* region that also extends to the *UTY* gene, and frequent variation in the *RBMY* and *PRY* multicopy gene families, was presented.

In the same year, a study of Y-CNVs inferred from sequence data in samples from the 1000 Genomes pilot phase (Espinosa et al. 2015) was published. The sample set consisted of 70 males from four populations (YRI, CEU, CHB and JPT) sequenced at 2.3× average depth; ten samples at variable depth, obtained by merging sequencing data from subsets of the same males belonging to the same haplogroup; and eight samples from the Complete Genomics Public Data set (v36 v2.0.0), at high (25.4×) depth. CNVs were mainly identified using a custom sequencing depth analysis, where the threshold and window size to be used were fine-tuned by comparing the full data for the reference sample (NA12891 from the CEU populations) to subsets of data from the same sample, varying said parameters and assuming that no CNV should be discovered in this case. To account for uncertainty in breakpoint definition, variants were merged if separated by 5 kb or less. This approach was complemented by the analysis of paired-end data available for some of the samples, and SNP array data and PCR amplifications were used to validate the full set; two variants discovered, but not validated, in previous studies (Mills et al. 2011; The 1000 Genomes Project Consortium 2012) were also validated and included in the final set. In total, 19 CNVs were reported, with 12 of these (63%) overlapping segmental duplication: again, repetitive regions appeared to be involved in the majority of rearrangements. A bias was observed towards the detection of larger events, as well as towards deletions over duplications. The samples in this study belonged to ten different Y haplogroups; by leveraging the univocal phylogeny available for the Y chromosome, the minimum number of mutational events for each CNV was determined: out of the 19 variants, four appeared to be caused by single events, while 15 appeared to be due to multiple mutations. A possible explanation of this imbalance is the different contribution of mutational mechanisms involved in CNV formation, namely non-homologous end joining (NHEJ) and NAHR, with homology-mediated mechanisms being more prone to recurrent events than non-homology mechanisms. Alternative allele (i.e. non-reference allele) count varied between one and 64; most of the variants were located in ampliconic or heterochromatic regions (8/19 and 7/19, respectively), with the latter being also associated with most of the high alternative allele counts. Six CNVs overlapped with members of five gene families on the chromosome (*BPY*, *CDY*, *DAZ*, *PRY*, *TSPY*). Common variants known to be present in the analysed populations (Jobling et al. 1996; Oakey and Tyler-Smith 1990; Redon et al. 2006; Tyler-Smith et al. 1988) were all observed in this study, showing that NGS data, even at low depth, can be successfully used to investigate Y-CNVs.

**Fig. 3:**
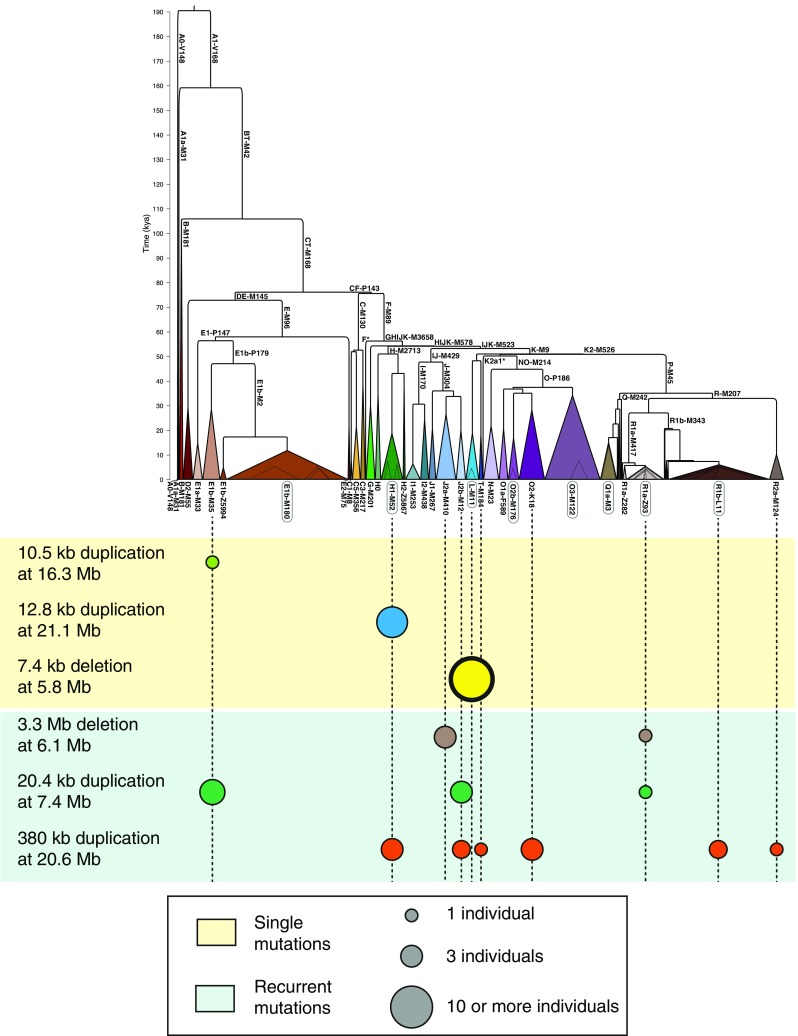
Numbers of CNV mutational events inferred from the phylogenetic tree. **a** Phylogeny based on the 1000 Genomes Project phase 3 data (from Poznik et al. 2016). **b** Examples of single mutational event CNVs (*light yellow background*) and multiple event CNVs (*light green background*). The 7.4 deletion at 5.8 Mb CNV, with a thicker surround, indicates that all sampled members of this haplogroup carry this CNV, while in the other examples only some member(s) of the haplogroup carry the CNV. For each listed CNV, approximate chromosomal position in GRCh37 is given by, e.g. ‘at 16.3 Mb’

In its third and final phase, the 1000 Genomes Project increased its samples size to 2504 individuals from 26 populations, and its mean whole-genome depth to 7.4× (The 1000 Genomes Project Consortium 2015). These resources enabled a large Y chromosome study to be carried out, which represents the widest description of MSY variation so far (Poznik et al. 2016). This work tackled all aspects of the MSY diversity: sequencing data for 1,244 males were used to discover over ~60,000 SNPs, which then were used to reconstruct an extensive phylogenetic tree; variants in other classes, including indels and multiple nucleotide polymorphisms (MNPs), CNVs and short tandem repeats (STRs), were discovered as well, and projected onto the high-resolution phylogeny to investigate their mutational patterns and properties. The main discovery method for CNVs was again the analysis of sequence data, using Genome STRiP (Handsaker et al. 2015), which infers structural rearrangements using the full information available from population-scale sequence data: local read depth variation, abnormal paired-end insert length, breakpoint-spanning reads, allele and haplotype sharing between samples, population heterogeneity caused by variant alleles, and negative correlation between alternative alleles (referred to as “allelic substitution”) (Handsaker et al. 2011). This approach was complemented by array CGH data, which were used to validate the Genome STRiP set, and call additional variants, for a total of 121 CNVs reported (100 in the Genome STRiP set only). A set of variants was validated using alkaline lysis fibre-FISH and molecular combing fibre-FISH, together with karyotyping for samples showing sex chromosomes aneuploidies. The unbiased phylogeny reconstructed in the study was leveraged to count the minimum number of mutational events for each locus (Fig. 3): the majority of variants were explained as single mutational events, although a few loci showed evidence for a high number of mutations; there was a higher prevalence of duplications compared to deletions. The presence of repetitive elements near putative breakpoints did not appear to be associated with highly mutable loci, although it appeared to be associated with longer variants, similar to observations on the autosomes (Conrad et al. 2010) (Fig. 1). Unsurprisingly, CNVs were predicted to have larger phenotypic effect than single nucleotide variants, as inferred from overlap with protein-coding genes: however, deletions overlapping protein-coding genes appeared to be more abundant than duplications overlapping protein-coding genes, while in a reanalysis of 1000 Genomes Project autosomal data (Sudmant et al. 2015) this relation appeared to be reversed. In other words, Y genes appeared to be more tolerant to deletions than autosomal genes, despite the haploidy of the chromosome, probably owing to their presence in multiple copies in many cases.

## Conclusions

The view of a Y chromosome confined to determining male fertility is being gradually superseded (Hughes and Page 2015), although the full understanding of copy number variation mechanisms and its impact on human biology is far from complete (Huddleston and Eichler 2016). Targeted studies, especially related to fertility, will likely continue to be carried out, perhaps on larger, population-specific cohorts, towards a complete description of variation in complex regions such as the *AZF* loci. Meanwhile, chromosome-wide studies should continue to uncover the full structural variation on the Y, filling the gaps and describing mutational mechanisms. It seems likely that almost all euchromatic Y-CNVs larger than 20 kb in the MSY that are frequent or fixed in any haplogroup have already been detected. Nevertheless, vast numbers of smaller and rarer Y-CNVs undoubtedly remain to be discovered, and study of the highly repeated highly variable heterochromatic segments has barely begun (Mathias et al. 1994). Future directions for Y-CNV investigations seem to lead towards the integration of different methods, especially with the developments of the long read technologies. A first attempt of such integration has been used to assemble the sequence of the gorilla Y chromosome (Tomaszkiewicz et al. 2016). This project employed flow-sorting (Dolezel et al. 2012) to obtain ~12,000 copies of the gorilla Y. These were used in a combination of short- and long-insert short read (Illumina Paired-End and Illumina Mate Pair sequencing, respectively) and long read sequencing (PacBio). Specific computational approaches were developed to increase the detection of Y-specific reads, which were used for a multi-step *de novo* assembly. RNA-seq from testis was employed to refine gene identification, and the size of ampliconic gene families was estimated using droplet digital PCR (Hindson et al. 2011). At least, until technologies leap forward once more, allowing cheap and accurate sequencing of Mb-sized molecules, complementing the weaknesses of one approach with the strengths of another is an attractive way to go.

## Box 1. The shadowing effect

Methods based on mapping data to the reference genome are affected by a limitation called “shadowing”. This phenomenon affects both intensity data, such as those produced by an array CGH experiment, and sequencing data, where the read depth interpretation is affected. Shadowing is caused by the presence of highly similar intra-chromosomal or inter-chromosomal duplicated sequences in the reference genome, and as such, is particularly relevant in repeat-rich regions. In such duplicated regions, experimental data map equally well to each of the reference copies. When a duplication or deletion of one of the copies occurs, the increase or decrease in signal will be averaged across all the reference copies. This means that the copy number variation will then be harder to identify, as it will result in a reduced signal difference compared to the copy number variation of a unique region; moreover, it will be impossible to tell which of the multiple copies in the reference genome has been duplicated or deleted. On the Y chromosome, shadowing is particularly relevant in ampliconic sequences, especially in palindromes, and in X–Y duplicated regions.
